# Self-medication and ILI etiologies among individuals presenting at pharmacies with influenza-like illness: Guatemala City, 2018 influenza season

**DOI:** 10.1186/s12889-022-13962-8

**Published:** 2022-08-13

**Authors:** Brooke M. Ramay, Jorge Jara, Maria Purificación Moreno, Patrizia Lupo, Carlos Serrano, Juan P. Alvis, C. Sofia Arriola, Vic Veguilla, S. Cornelia Kaydos-Daniels

**Affiliations:** 1 Program for Influenza and Other Respiratory Viruses, Center for Health Studies, Research Institute, Universidad del Valle de Guatemala (UVG), Guatemala City, Guatemala; 2 U.S. Centers for Disease Control and Prevention (CDC), Atlanta, USA

**Keywords:** Influenza Like Illness, Antibiotics, Pharmacy based study

## Abstract

**Objectives:**

We aimed to characterize the proportion of clients presenting to community pharmacies with influenza-like illness (ILI) and the severity of their illness; the proportion with detectable influenza A, influenza B, and other pathogens (i.e., parainfluenza I, II, and III, adenovirus, respiratory syncytial virus, human metapneumovirus); and to describe their self-medication practices.

**Methods:**

A cross-sectional study was conducted in six pharmacies in Guatemala City. Study personnel collected nasopharyngeal and oropharyngeal swabs from participants who met the ILI case definition and who were self-medicating for the current episode. Participants were tested for influenza A and B and other pathogens using real-time RT-PCR. Participants’ ILI-associated self-medication practices were documented using a questionnaire.

**Results:**

Of all patients entering the pharmacy during peak hours who responded to a screening survey (*n* = 18,016) 6% (*n* = 1029) self-reported ILI symptoms, of which 45% (*n* = 470/1029) met the study case definition of ILI. Thirty-one percent (148/470) met inclusion criteria, of which 87% (130/148) accepted participation and were enrolled in the study. Among 130 participants, nearly half tested positive for viral infection (*n* = 55, 42.3%) and belonged to groups at low risk for complications from influenza. The prevalence of influenza A was 29% (*n* = 35). Thirteen percent of the study population (*n* = 17) tested positive for a respiratory virus other than influenza. Sixty-four percent of participants (*n* = 83) reported interest in receiving influenza vaccination if it were to become available in the pharmacy. Medications purchased included symptom-relieving multi-ingredient cold medications (*n* = 43/100, 43%), nonsteroidal anti-inflammatory drugs (*n* = 23, 23%), and antibiotics (*n* = 16, 16%). Antibiotic use was essentially equal among antibiotic users regardless of viral status. The broad-spectrum antibiotics ceftriaxone and azithromycin were the most common antibiotics purchased.

**Conclusions:**

During a typical influenza season, a relatively low proportion of all pharmacy visitors were experiencing influenza symptoms. A high proportion of clients presenting to pharmacies with ILI tested positive for a respiratory virus. Programs that guide appropriate use of antibiotics in this population are needed and become increasingly important during pandemics caused by respiratory viral pathogens.

**Supplementary Information:**

The online version contains supplementary material available at 10.1186/s12889-022-13962-8.

## Introduction

Influenza is a vaccine-preventable viral disease that causes significant morbidity and mortality worldwide [1]. Influenza-like illness (ILI) is characterized by acute onset of fever, cough, and a range of other possible symptoms such as headache, myalgia, nasal congestion, fatigue, chills, and sore throat. Management of symptoms using medications widely available over-the-counter at pharmacies, is usually sufficient for mild influenza. While non-severe cases of influenza are usually self-limiting, ILI symptoms can be difficult to distinguish, leading to confusion between viral respiratory infections and other differential diagnoses that may require other treatments. Although it is well established that inappropriate medication use can result in unnecessary side effects and increase direct costs to patients, there is little to no information on the proportion of patients that visit pharmacies with ILI to self-treat symptoms [2]. Self-medication with antimicrobials for ILI and the adverse effects of inappropriate self-care are a dangerous combination that may be interrupted through pharmacy-based interventions [3–5].

Pharmacy based interventions that support prevention, detection and treatment of influenza are beneficial in high-income countries for improving vaccination rates, and facilitating appropriate guidance for symptomatic care, but pharmacy based interventions are uncommonly implemented in low- to middle-income countries (LMIC) [3]. The role of pharmacies have become increasingly apparent during the COVID-19 pandemic where pharmacists have contributed to improve supply chain management, workforce development, testing and vaccination [6–9]. Highly trained professionals work closely, and in some cases under protocols, with physicians to guide prevention strategies and rational use of medications [10]. In most low-income countries, however, pharmacy attendants receive little to no training in medication use, and consequently, prevention and treatment strategies encounter several barriers toward implementation [11]. Notable pharmacist based interventions from the United States and other high income countries have taken decades to achieve and will require concerted efforts in LMICs to achieve significant impact [12–14].

A significant proportion of patients with mild influenza are thought to seek care primarily at private pharmacies in Guatemala; thus, these settings may be appropriate for ILI detection, influenza prevention through vaccination campaigns, and medication stewardship [15, 16]. Medications are provided free of charge to the majority of the population who seek care at the ministry of health (MOH), yet true *access* to medications through the MOH is limited due to frequent stock-outs. Most medications, however, are available for out-of-pocket purchase at private pharmacies where there are few dispensing restrictions. Oseltamivir, corticosteroids, and multi-ingredient cold medications are sold without prescriptions at public and private pharmacies [17]. At the time this study was carried out, March–July, 2018, there were no restrictions requiring a prescription for antibiotic purchase and this study provides historical information about medication use before legislation was passed.

In order to explore the feasibility and impact of a pharmacy-based ILI intervention, this study aimed to (1) characterize the proportion of clients presenting to community pharmacies in Guatemala City, Guatemala with self-reported ILI; (2) to detect the proportion of that population infected with influenza A or B and other viral respiratory pathogens using real-time reverse-transcriptase polymerase chain reaction (rRT-PCR) among individuals with ILI, and (3) to describe self-medication practices among those with ILI symptoms.

## Methods

### Study design, population, and sample size

We conducted a cross-sectional exploratory study from March 13th to July 31st, 2018, in six private pharmacies, from a total of 5581 registered pharmacies in Guatemala. Pharmacies were selected from a range of neighborhoods geographically distributed across Guatemala City during the predicted 2018 influenza season in Guatemala (epidemiological weeks 11–31). Pharmacies belonging to the same private pharmacy chain were chosen based on the availability of private areas for participants’ screening and sample collection, well-defined peak hours (determined by pharmacy marketing data), and serving customers of high, middle, and middle-low socio-economic status.

An estimated 8608 clients were projected to visit study pharmacies during the study hours (Monday through Friday, 3–8 pm) per pharmacy marketing data. Sample size was determined to capture clients entering the pharmacy with self-reported ILI. Due to lack of any prior data, we calculated the sample size for an unknown population proportion, with 5% margin of error, 95% confidence interval, and maximized the error for proportion, estimating that 50% of pharmacy clients would present with ILI. We determined that a sample size of 356 participants would be adequate to describe the population with ILI presenting to the selected pharmacies during peak hours.

### Screening, enrollment, samples taken

Participants were enrolled from 3 to 8 pm to screen the highest number of clients possible. Trained field technicians approached every client who entered the pharmacy; those who confirmed having “a cold” or “the flu” were invited to hear more about the study by study-physicians/study-pharmacists and taken through the ILI screening questions. Parents/guardians answered on behalf of study participants ≤7 years. All adult participants provided written informed consent, participants 7–17 years old provided informed assent, and parents/guardians consented for participants < 7 years old.

Participants were included if they had confirmed ILI, defined as self-reported history or measured fever ≥38 °C with the presence of cough and/or sore throat, with onset ≤10 days before the pharmacy visit; and if they were seeking self-medication for their current ILI episode.

The following clients were excluded from participation: those who had been prescribed medication by a physician, those who presented to the pharmacy for reasons other than seeking medications for ILI symptoms, patients using phone-in pharmacy delivery services, patients using drive-through pharmacy services, individuals who arrived to purchase medications for persons not present, and children < 5 years old showing any general danger sign according to Integrated Management of Childhood Illness guidelines (IMCI: a child who is unable to drink or breastfeed; who is vomiting in a quantity sufficient to vacate the stomach completely; or who has had a convulsion) [18]. Further, clients with danger signs for respiratory illness were not enrolled in the study but rather instructed to seek care immediately at the nearest health facility. These are, adults presenting with respiratory rate greater than 26 breaths/minute or blood O2 saturation < 90% while breathing ambient air (measured by digital pulse oximetry); among pregnant women, the danger sign is considered blood O2 saturation < 95% while breathing ambient air [19]. High risk individuals were defined as those with asthma, diabetes, chronic cardiac disease, chronic renal disease, chronic liver disease, chronic neurological disease, a chronic hematological disorder, obesity, or tuberculosis, or who were pregnant or older than age 65 [20]. Additional, country specific, high risk categories were included to account for locally relevant chronic conditions. These are: thyroid disease and stunted growth [20].

A total of three swabs (two nasopharyngeal and one oropharyngeal sample) were collected by trained personnel from participants to use in two separate laboratory tests: an antigen-based rapid influenza test and rRT-PCR analysis for viral respiratory diseases. One nasopharyngeal sample was immediately tested at the pharmacy by rapid influenza test using the SD BIOLINE Influenza Ag A/B/A(H1N1) pandemic® test. Participants were informed of their rapid-test results after completing all portions of the questionnaire. The remaining nasopharyngeal and oropharyngeal swabs were placed into universal transport media and stored in pharmacies at 4–8 °C overnight before being picked up the following day (samples were stored at pharmacies for ≤24 hours). Samples were transported at 4–8 °C to the Center for Health Studies-Universidad del Valle Guatemala (CES-UVG) laboratory for viral analysis. At CES-UVG, samples were immediately cold-centrifuged, aliquoted, and stored at − 80 °C. Genetic material was extracted within 7 days using QIAamp MinElute Virus Spin Kit. Extracted DNA and RNA were processed for viral presence using rRT-PCR.

Nasal and oropharyngeal swabs were tested by singleplex rRT-PCR at the CES-UVG laboratory according to US Centers for Disease Control and Prevention (CDC) protocols for respiratory syncytial virus (RSV), parainfluenza viruses (PIV) 1–3, adenovirus, human metapneumoviruses (hMPV), rhinoviruses, and influenza viruses, including influenza A(H1N1) pdm09, A(H3N2), and B. *S. pneumoniae* was extracted using a PCR mix for multiplex assay lytA-CDC; *S. aureus* was identified using the nuc-gene [21–23]. Study personnel provided information to participants to contact lab personnel to inquire about their results at their convenience, at least 1 week after study participation.

### Self-medication practice questionnaires

Trained physicians and a pharmacist administered the questionnaire to enrolled participants using smartphone Android devices and Open Data Kit (ODK), data were stored on the Center for Health Studies secure server. The questionnaire consisted of 32 questions in three categories: participant demographics and specific underlying conditions associated with influenza complications, ILI symptoms (including body temperature measurement), and self-medication practices including vaccination knowledge/interest. Participants indicated their average monthly income by selecting their income category. These categories were created based on the minimum wage in Guatemala (410USD/month for non-agricultural workers [24]) and ranged from less than 256USD to greater than 1300USD (using a 7.8 USD to conversion rate to local currency).

ILI-associated medication purchases of each study participant were documented by taking photos of receipts during the course of the study encounter. Medications were coded into 7 medication classes: multi-ingredient cold medication with analgesics, multi-ingredient cold medication without analgesics, nonsteroidal anti-inflammatory drugs (NSAIDs), antibiotics, single-ingredient medications, vitamins, and natural medications. Medication purchase timing varied with rapid-test results: on some occasions participants were given rapid-test results before medication purchase, and sometimes after medication purchase. This study was not designed to detect influences on medication purchase according to rapid-test results. Patients were interviewed after medication purchase to record medication type and cost.

### Data analysis

We used STATA [25] to produce descriptive statistics on demographics, viral etiologies, and self-medication practices. We report frequencies of categorical data, and median and interquartile ranges [IQR] for continuous variables.

### Ethics approval

This study was conducted in accordance with all applicable local regulatory requirements and the principles of the Declaration of Helsinki. Protocol number 171–09-2017 was approved by the Universidad del Valle, Center for Health Studies (UVG- CHS) Ethics Research committee. Study personnel, including 5 physicians and 1 pharmacist were trained to carry out consent, data and sample collection prior to initiating the study. All adult participants provided written informed consent, participants 7–17 years old provided informed assent, and parents/guardians provided informed consent for participants 7–17 years old.

## Results

**Fig. 1:**
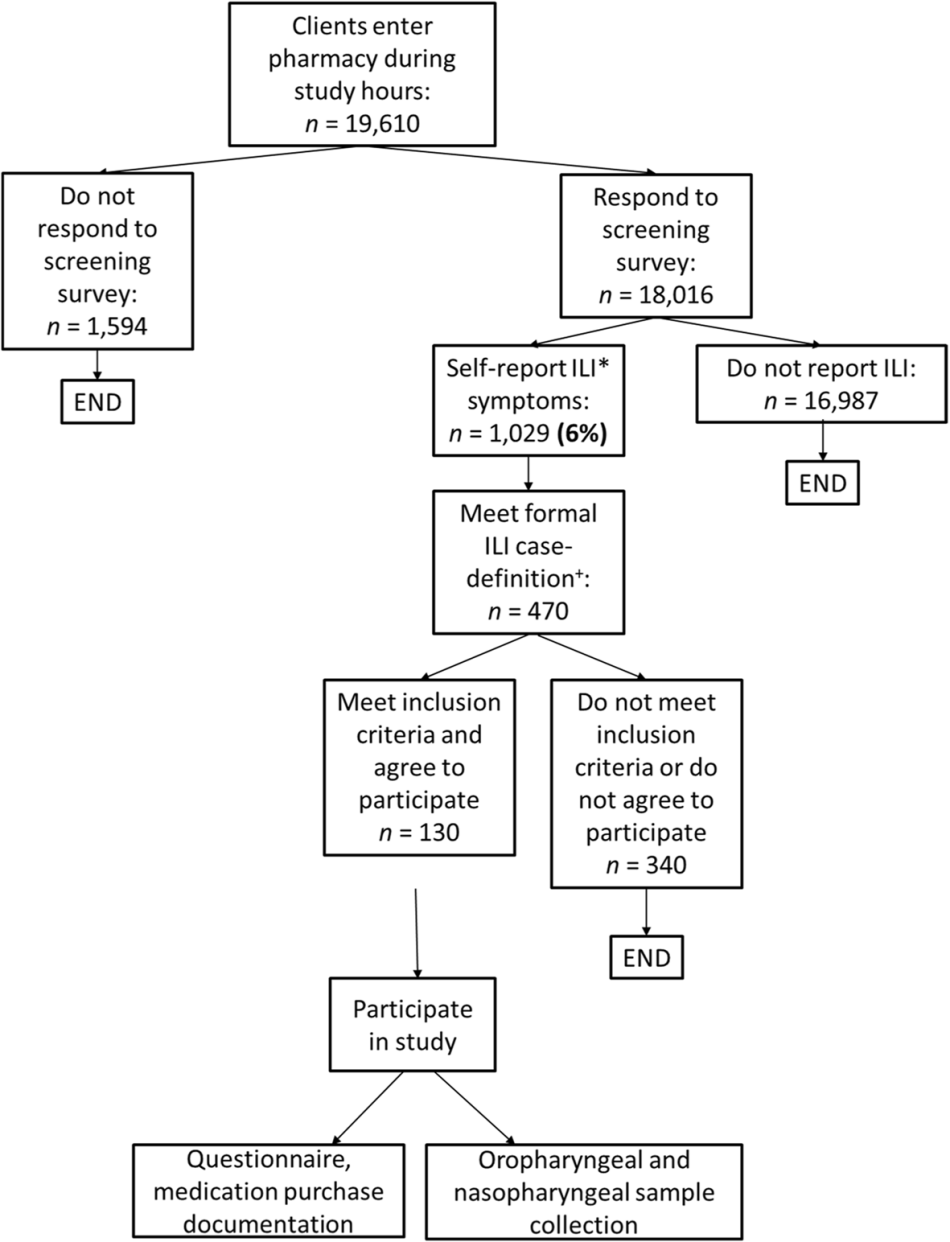
Study recruitment and participation. Of 19,610 clients approaching study-pharmacies, 130 met the inclusion criteria and accepted participation. ^*^Self-reported influenza like illness (ILI): Participants were initially asked if they had a “cold” or “flu” to identify self-reported ILI. ^+^Formal ILI-case definition, defined through screening questionnaire: self-reported history or measured fever ≥38 °C with the presence of cough and/or sore throat, with onset within the previous 10 days

A total of 19,610 clients visited study pharmacies during peak hours, of whom 92% (18,016) responded to the self-report ILI survey (Fig. 1). Six percent (*n* = 1029) of respondents self-reported “cold” or “flu-like” symptoms. Sixty five percent (667/1029) participated in the screening process to establish eligibility for enrollment. Seventy percent (*n* = 470/667) of those who went through the screening process met the study case-definition of ILI: 12% (*n* = 53) reported a fever and sore throat; 11% (*n* = 49) reported a fever and cough; and 77% (*n* = 363) reported a fever, cough, and sore throat. Sixty eight percent of participants were excluded from the study (322/470).

Reasons for exclusion were as follows: 4 (1%) children were not accompanied by an adult, 1 baby (0.2%) showed two general danger signs according to IMCI (vomiting to the point of emptying stomach completely and difficulty breathing), and 2 (0.4%) clients had blood oxygen saturation < 90% while breathing ambient air. Fifty-eight percent of excluded clients (*n* = 274) were not purchasing medications for the current illness, and 9% (*n* = 41) were purchasing medications with a physician’s prescription. A total of 148 participants were taken through informed consent, of which 87% (130/148) accepted participation.

### Participant characteristics

The median age of study participants was 32 years (IQR: 25–47), of whom 59% (*n* = 77) were women, 54% (*n* = 70) reported obtaining a university degree, and 10% (*n* = 12) were health care workers. Twenty-nine percent (*n* = 38) of participants reported comorbidities (including age and pregnancy) that fit the criteria for population at risk to have complications due to influenza. Sixty-nine percent (*n* = 90) of participants reported having medical insurance. Of these, 38% (*n* = 49) reported private insurance, 30% (*n* = 39) participants reported medical coverage by the Guatemalan social security system, and 2% (*n* = 2) participants reported coverage through the national military service. Participants reported their monthly income as follows: 13% (*n* = 17) reported incomes <$267, 52% (*n* = 68) reported income ranges of $268–$1333, 21% (*n* = 27) reported an income >$1334; and 14% (*n* = 18) did not report their income (see Table 1).

**Table 1:** Participant demographic characteristics

Variables	Categories	***n*** = 130	%
**Sex**	Female	77	59%
**Pregnant, and over 18 years old**	No	68	94%
	Yes	2	3%
	No response	2	3%
**Age group**	< 5 years	0	0%
	5–14 years	7	5%
	15–49 years	98	75%
	50–64 years	18	14%
	≥65 years	7	5%
**High-risk group for severe illness**^a^**(*n*** **= 49 illnesses)**	Asthma	12	24%
	> 65 years old	7	14%
	Diabetes	7	14%
	Stunted growth	5	10%
	Thyroid disease	4	8%
	Other^b^	14	29%
**Ethnicity**	Ladino	113	87%
	Indigenous	6	5%
	White	3	2%
	Asian	1	< 1%
	Doesn’t know	7	5%
**Education**	Higher education	70	54%
	Secondary education	35	27%
	Primary education	22	17%
	No formal education	2	2%
	No response	1	< 1%
**Health insurance**	Private health insurance	49	38%
	Social Security insurance	39	30%
	Military insurance	2	2%
	None	40	31%
**Interviewed participant was a health care worker**	No	105	90%
	Yes	12	10%
**Monthly income range (in USD)**	<$267.00	17	13%
	$268.00–$1333.00	68	52%
	>$1334.00	27	21%
	No response	18	14%

^a^Among 38 people in high-risk groups, 8 reported 2 illnesses, 1 reported 3 illnesses, and 1 reported 4 illnesses; a total of 49 illnesses were reported

^b^Cardiopathies (*n* = 2), pregnancy (*n* = 2), cystic fibrosis (*n* = 1), history of stroke (*n* = 1) history of chronic kidney disease (*n* = 1), chronic hematological disorder (*n* = 1), neurological disorder (*n* = 1), bronchopulmonary dysplasia (*n* = 1), history of febrile seizures (*n* = 4)

### Presence of viral pathogens

Ninety-six percent (*n* = 125) of study participants provided a nasopharyngeal sample for the rapid test and rRT-PCR. Fifteen percent (*n* = 17) tested positive for influenza A, according to the rapid test. Of the 130 participants providing rRT-PCR samples, 29% (*n* = 38/130) tested positive for influenza with only one virus/strain: 66% (*n* = 25/38) for influenza A(H3N2), and 34% (*n* = 13/38) for influenza A(H1N1). In 13% of participants (*n* = 17/130), other viral respiratory pathogens were detected: 5% parainfluenza III (*n* = 6), 3% parainfluenza I (*n* = 4), 2% parainfluenza II (*n* = 3), adenovirus 2% (*n* = 2), 1% human metapneumovirus (*n* = 1), 1% respiratory syncytial virus (*n* = 1%) (Table 2).

**Table 2:** Presence of laboratory-confirmed virus

	***n*** = 130	%
**Influenza A**	**38**	**29% (38/130)**
Influenza A (H3N2)	22	58% (22/38)
Influenza A (H1N1pdm09)	13	34% (13/38)
Influenza A (H3N2 + RSV)	1	3% (1/38)
Influenza A (H3N2 + Adenovirus)	1	3%(1/38)
Influenza A (H3N2 + Parainfluenza III)	1	3%(1/38)
**Positive viral result, other than Influenza A**	**17**	**13%(17/130)**
Parainfluenza III	6	35%(6/17)
Parainfluenza I	4	24%(4/17)
Parainfluenza II	3	18%(3/17)
Adenovirus	2	12%(2/17)
Human metapneumovirus	1	6%(1/17)
**Negative viral result**	**75**	**58% (75/130)**

Note The proportion of confirmed cases for influenza A during the peak flu season in Central America confirmed a relatively high prevalence of influenza A. Nasal and oropharyngeal swabs were tested by singleplex rRT-PCR according to US Centers for Disease Control and Prevention protocols for respiratory syncytial virus (RSV), parainfluenza viruses (PIV) 1–3, adenovirus, human metapneumoviruses (hMPV), rhinoviruses, and influenza viruses, including influenza A/H1N1pdm09, A/H3N2, and B (Morgan et al., 2013; Pan American Health Organization, 2011)

### Self-medication and influenza vaccination

Among the 130 participants that presented to self-medicate for ILI symptoms, 78% (*n* = 100) provided receipts of medication purchases. In some cases, participants purchased more than one medication category. The most common medication purchases were multi-ingredient cold medications (54%, *n* = 54), natural medications and vitamins (26%, *n* = 26), non-steroidal anti-inflammatory drugs (NSAIDs; 23%, *n* = 23), other medications (Inhaled bronchodilators, glucocorticoids, methocarbamol (muscle relaxant), topical analgesics, cough drops) (18%, *n* = 18), antibiotics (16%, *n* = 16), and single-ingredient medications (antihistamines, expectorants, and decongestants) (*n* = 12, 12%). Antibiotics purchased for self-medication included azithromycin 25% (*n* = 4); ceftriaxone 25% (*n* = 4); amoxicillin with clavulanic acid 19% (*n* = 3); amoxicillin 13% (*n* = 2); chloramphenicol 6% (*n* = 1); dicloxacillin 6% (*n* = 1); and tetracycline 6% (*n* = 1). The type of antibiotic purchased and rRT-PCR–confirmed viral respiratory result can be found in Table 3. Of 16 participants purchasing antibiotics, 56% (*n* = 9) had a positive viral result. Interestingly, broad spectrum antibiotic use was similar in participants regardless of viral status. Azithromycin was used in 22% (*n =* 2/9) versus 29% (*n =* 2/7) of viral, versus non-viral cases respectively. Further, ceftriaxone was used in 22% (*n =* 2/9) versus 29% (*n =* 2/7) of viral versus non-viral cases respectively. The median amount spent per patient on self-medication was $4 (IQR: $2–9). Antiviral medications, including oseltamivir were not purchased by any participants.

**Table 3:** Presence/absence of virus per rRT-PCR and antibiotic purchased (*n* = 16)

Virus identified via rRT-PCR	Antibiotic purchased
Influenza A H1N1 Pdm09	Amoxicillin/clavulaunic acid (*n* = 1)
Influenza A H3N2	Amoxicillin/clavulaunic acid (*n* = 1)
Influenza A H3N2+ Adenovirus	Chloramphenicol (*n* = 1)
Influenza A H3N2+ Parainfluenza III	Ceftriaxone (*n* = 1)
Adenovirus	Ceftriaxone (*n* = 1)
	Tetracycline (*n* = 1)
Parainfluenza I	Azithromycin (*n* = 2)
Respiratory sinicital virus	Amoxicillin/clavulaunic acid (*n* = 1)
Negative viral result	Ceftriaxone (*n* = 2)
	Amoxicillin (*n* = 2)
	Azithromycin (*n* = 2)
	Dicloxacillin (*n* = 1)

Note 56% of participants purchasing antibiotics had a positive viral result (*n* = 9). The broad-spectrum antibiotics ceftriaxone and azithromycin were the most commonly purchased. PCR viral results were made available to participants after medication purchase

Of 129 respondents who completed the questionnaire (99%, 129/130), 5% (*n* = 6) reported receiving influenza vaccination for the current season. Four of the six respondents (66%) were in a high-risk group for influenza vaccination in Guatemala: one was > 65 years old with a chronic condition, one was a health care provider, and two had chronic conditions putting them at risk for complications due to influenza. Thirty-four of 38 participants in target groups for influenza vaccination did not receive the influenza vaccine (89%). Sixty-four percent of participants (*n* = 83) reported interest in receiving influenza vaccination if it were to become available in the pharmacy. The highest proportion of respondents (40%; 51/129) were willing to pay $7–$21 for vaccination, 29% (*n* = 37) were willing to pay nothing or only a minimal amount (from $0.00–$7), and 28% (*n* = 36) were willing to pay more than $21.00 for vaccination.

## Discussion

We carried out a cross-sectional study in private pharmacies in Guatemala City during peak influenza seasons in 2018, March–July. Six percent of pharmacy clients presented with self-reported ILI symptoms. Of enrolled study participants, almost half had a positive respiratory viral result. Estimates of the prevalence of influenza are similar to other community household studies, where anywhere from 15 to 35% of the population presenting with ILI symptoms have a positive influenza result via rRT-PCR [26, 27]. The majority of participants in our study belonged to groups at low risk for complications from influenza, and purchased a wide range of medications, from antibiotics to corticosteroids and muscle relaxants. Identifying the number of ILI patients who visit pharmacies to self-treat ILI, and the ability to describe the severity of their illness, is an important first step toward development of pharmacy- based interventions. This becomes important during outbreaks of respiratory viral pathogens when pharmacies tend to report higher patient volumes and are well positioned to serve the community at a broader capacity through vaccination and testing [28]. Here, we show that the majority of participants demonstrated interest in receiving the influenza vaccine if it became available in the pharmacy and would be willing to pay seven dollars or more, further justifying establishment of pharmacy based interventions.

Mild cases of influenza, or patients who typically don’t seek hospital care, may be identified through community household studies, but these studies are costly, time-consuming, and provide information from a specific subset of the community which is further complicated in regions with variable influenza seasons [27]. Pharmacies on the other hand may be a harbinger for public health practitioners tracking ILI trends, especially at the beginning of the flu season, or early in an epidemic of other respiratory pathogens [29]. Guatemala’s current influenza surveillance program, aligned with the Pan American Health Organization and World Health Organization, is primarily hospital-based (World Health Organization, 2013) and focuses on severe cases of influenza. Milder, self-limiting cases likely represent a large proportion of the population affected by influenza and remain undetected by local surveillance [26, 30, 31]. Pharmacy-based programs offering rapid tests to patients to identify influenza and group A streptococcus have improved access to diagnostic tools, particularly for patients without a primary care provider [10, 32]. During the COVID-19 pandemic, pharmacists have delivered responses including COVID-19 testing, vaccinations, providing health information related to COVID-19, and support point-of-care testing for chronic disease in times when clinical services were limited at physicians’ offices [33, 34].

Our results suggest that participants use broad-spectrum antibiotics to self-treat ILI. Similar rates of antibiotic use for ILI were reported in one large Peruvian sentinel surveillance program, in which nearly 15% of participants reported antibiotic use for ILI symptoms, with one-fourth having documented influenza viral infection [35]. Self-medication with antibiotics occurs in many low- to middle-income countries, particularly for self-treating ILI symptoms [36–39]. Because antibiotic sales are not regulated, antibiotics are also sold without a prescription in supermarkets, open-markets and corner stores (or “tiendas”) [40]. Legislation was passed in Guatemala (August 2019) requiring a prescription for the sale of antibiotics (“Acuerdo Ministerial Número 145-2019, Ministerio de Salud Publica y Asistencia Social, Guatemala,” 2019) but several barriers to policy implementation have surfaced including: 1) support for implementation and enforcement of the ministerial accord and 2) public debate surrounding access to health care prescription services. Unsupervised antimicrobial self-medication is frequently associated with inappropriate drug use and should be discouraged regardless of a positive or negative laboratory viral result.

We provide evidence suggesting an increase in the prevalence of broad-spectrum antibiotic use in patients with more severe cases of ILI. In previous pharmacy-based studies in Guatemala, self-medication with antibiotics was common, occurring in over 75% of study population across socio-economic sectors. In this general population, however, the most common antibiotics selected for self-medication included amoxicillin and tetracycline [16]. Use of broad-spectrum antibiotics drives selection pressure for antimicrobial resistance. For example, azithromycin (a broad-spectrum macrolide) is associated with increased carriage rates of *S. aureus* and with macrolide-resistant *S. pneumoniae* and *S. aureus* that are both cumulative and dose dependent [41]*.* Ceftriaxone, an intramuscular broad-spectrum beta-lactam also purchased by our study population, is commonly used in the hospital-*inpatient* setting with caution as to not promote strains of extended-spectrum beta lactamase resistance [42, 43]. Antibiotic misuse is predicted to cause major long-term effects on health care outcomes and spending. In low- to middle-income countries, estimations of the proportion of antibiotic-resistant strains of bacteria are high, and the cost required to implement interventions to prevent resistance are much greater [44, 45]. Systematic guidance in the rational use of antibiotics in patients self-medicating for ILI may be a useful strategy to moderate antibiotic use as these new regulations are rolled out in the community pharmacy setting. This becomes increasingly relevant during pandemic preparedness when pharmacy professionals may provide guidance to treat symptoms to avoid overburdened health systems including emergency rooms and health care centers.

Although we provide important evidence for the prevalence of influenza in neighborhoods served by these community pharmacies, there are some limitations to our study. First, because we aimed to describe self-medication practices, we excluded many potential study participants who did not purchase medications for their ILI symptoms. Although participants were representative of the neighborhoods where the study was carried out, the overall income of enrolled participants was higher than the average income of Guatemala City residents. Nevertheless, approximately 65% of drug purchases are made in the private sector in Guatemala, increasing the impact findings from this private pharmacy study may have on current pharmacy policy and practice [46, 47]. Also, some patients were given their influenza rapid-test results before making a medication purchase which may have disproportionately affected their medication selection. Nevertheless, this is the first study that provides sound methodology that can be adjusted to include a broader range of patients and used to design future studies carried out in community pharmacies. Because our study was carried out during “peak hours” of pharmacies, we captured an estimated 40% of daily clientele, screening roughly 18,000 people. We underestimated the number of clients that would visit pharmacies yet underestimated the number of ILI patients who would meet inclusion criteria. In order to characterize risk factors of clients participating with ILI, sample sizes must be much larger so that differences between those with a positive and a negative viral result may be described.

## Conclusions

A high proportion of clients presenting at pharmacies In Guatemala City with ILI had a positive respiratory viral result. Participants at low risk for complications from influenza purchased a wide range of medications, from antibiotics to corticosteroids and muscle relaxants. Educational programs that effectively communicate the appropriate use of medications in this population are needed, particularly those that aim to reduce inappropriate use of antibiotics. Development of pharmacy-based policies for vaccine administration, and rational use of medications, including enforcing the requirement of a prescription for antibiotics, are important first steps toward initiating appropriate pharmacy-based interventions. These contributions impact public policy discussion surrounding the role of pharmacies in prevention, and rational use of medications for ILI in Guatemala and similar countries.

## Supplementary Information

**Additional file 1.** Geographic description of study pharmacy locations.

## Data Availability

The datasets used and analyzed during the current study available from the corresponding author on reasonable request.
