# Supplementary material for: Self-medication and ILI etiologies among individuals presenting at pharmacies with influenza-like illness: Guatemala City, 2018 influenza season
Source: BMC Public Health. 2022 Aug 13;22:1541. doi: 10.1186/s12889-022-13962-8 (PMC9374570; doi:10.1186/s12889-022-13962-8)
Supplement: Supplementary file 1 — Additional file 1. Geographic description of study pharmacy locations. [file 12889_2022_13962_MOESM1_ESM.docx]

| Pharmacy 1 | Located within a suburb of Guatemala City on the major freeway within this area and is 1 of 12 pharmacies within a 4km distance. The population of the suburb is 66,460 people. |
| --- | --- |
| Pharmacy 2 | Located on one of the major freeways leading to the port city on the Pacific Ocean in the southern region Guatemala City. Approximately 88,000 vehicles transit this freeway per day including urban, suburban, freight trucks and public buses. This is 1 of 3 pharmacies within a 4km distance on this road. |
| Pharmacy 3 | Located next to one of the largest shopping centers in Guatemala City and within 4 km of the largest tertiary public hospital in the country. |
| Pharmacy 4 | Situated in a semi-residential neighborhood between two zones with high rise apartment complexes in addition to office buildings. This pharmacy is 1 of 3 pharmacies on the major road that divides the line between two zones (3km distance) |
| Pharmacy 5 | Situated on the major road that crosses a residential neighborhood with a population of 14,500. This pharmacy is 1 in 7 along 4 km of the major road bisecting the zone. |
| Pharmacy 6 | Located in the largest in Guatemala City located in a shopping mall in the northern region. The shopping mall is an attraction for people who both reside and for people who live outside the zone. |

Supplement 1. Geographic description of study pharmacy locations
